# Generation of iPSC-Derived iNKT Cells with Pro-Hematopoietic Activity

**DOI:** 10.1007/s12015-025-11031-2

**Published:** 2025-12-11

**Authors:** Akhilesh Kumar, Sarah Ferguson, Saritha S. D’Souza, Nikhila S. Bharadwaj, Mathew Raymond, Jenny E. Gumperz, Igor I. Slukvin

**Affiliations:** 1 Wisconsin National Primate Research Center, University of Wisconsin-Madison, Madison, WI 53712 USA; 2 Department of Medical Microbiology and Immunology, School of Medicine and Public Health, University of Wisconsin, Madison, WI 53706 USA; 3 Department of Cell and Regenerative Biology, School of Medicine and Public Health, University of Wisconsin, Madison, WI 53707 USA; 4 Department of Pathology and Laboratory Medicine, School of Medicine and Public Health, University of Wisconsin, 1220 Capitol Court, Madison, WI 53792 USA

**Keywords:** iNKT cells, Induced pluripotent stem cells, Reprogramming, Myeloid progenitors, Hematopoiesis

## Abstract

Human invariant natural killer T (iNKT) cells are a conserved population of innate-like T cells that are activated by glycolipid antigens. In addition to their well-known role in anti-tumor function, iNKT cells are also involved in regulating and maintaining hematopoiesis in the bone marrow. Here, we present the reprogramming of human CD4^+^Vα24^+^Vβ11^+^ iNKT cells into induced pluripotent stem cells (iNKT-iPSCs) and describe a novel chemically defined, feeder-free 3D spheroid method for generating CD34^+^ cells from iNKT-iPSCs, followed by their re-differentiation into functional Vα24^+^Vβ11^+^ iNKT cells (i-iNKT) with pro-hematopoietic activity. The i-iNKT cells showed specific binding to CD1d tetramers loaded with the lipid antigen α-galactosylceramide and had a similar transcription factor profile to that of somatic CD4^+^ iNKT cells. Additionally, in response to CD3 stimulation, the i-iNKT cells produced cytokines with hematopoietic potential and promoted expansion/differentiation of myeloid progenitors. These findings suggest the feasibility of using iPSCs as off-the-shelf i-iNKT cell sources to enhance the hematopoietic activity of bone marrow after hematopoietic stem cell (HSC) transplantation.

**Supplementary Information:**

The online version contains supplementary material available at 10.1007/s12015-025-11031-2.

## Introduction

Invariant natural killer T (iNKT) cells are a discrete subset of human αβ T cells that express a semi-invariant TCR comprised of a canonically rearranged TCRα chain TRAV10-TRAJ18 (Vα24-Jα18) paired with diversely rearranged TRBV25^+^ (Vβ11^+^) TCRβ chains [1]. In contrast to conventional αβ T cells that recognize peptide antigens bound to classical MHC-encoded antigen presenting molecules, iNKT cells recognize lipids and glycolipids presented by non-classical CD1d antigen presenting molecules [2]. Moreover, unlike adaptive T cells, which start out in a naive state then become functionally specialized during the course of an immune response, iNKT cells undergo a distinct developmental program that is characterized by the expression of PLZF, a master-regulator transcription factor that confers an innate-like phenotype [3, 4]. As a result, iNKT cells do not require priming and rapidly carry out effector functions upon stimulation, including producing a wide variety of cytokines and mediating anti-tumor activity through cytolysis and indirect adjuvant-like mechanisms [3]. Additionally, iNKT cells constitutively carry out innate-like functions that include regulating hematopoiesis in the bone marrow [5, 6], and have been shown to profoundly enhance human hematopoietic engraftment in a murine xenotransplant model [7]. Based on their powerful, multi-functional immunological profile, iNKT cells have increasingly garnered attention as a potential platform for cellular immunotherapies [8, 9].

Because of the non-polymorphic nature of CD1d molecules and high conservation of lipid antigens, iNKT cells are ’donor unrestricted’ [10]. iNKT cells, therefore, do not mediate allogeneic responses and can be used for off-the-shelf immunotherapies without the risk of graft-versus-host diseases (GvHD) [11]. Several clinical trials have demonstrated the efficacy and tolerability of immunotherapy using either genetically unmodified or chimeric antigen receptor (CAR)- transduced iNKT cells for malignancies and COVID-ARDS [12–18]. Despite the rarity of iNKT cells in peripheral blood, they can be expanded at a large scale to generate products for clinical use. However, the presence of contaminating conventional T cells in the expanded iNKT product poses a risk for GvHD and may hamper their use [19, 20]. Allogeneic iNKT cells may also be susceptible to rejection by the host due to HLA mismatches. Thus, clinically adaptable methods for iNKT cell production from hypoimmunogenic cell sources are of interest.

Induced pluripotent stem cells (iPSCs) provide several advantages for the manufacturing of iNKT cells. They are easily amendable to multiple genetic editing, allowing the generation of hypoimmunogenic cells expressing molecules targeting cancer cells. Since iPSCs can be clonally selected and expanded indefinitely, it is feasible to generate off-the-shelf iNKT cell products with uniform gene editing [21]. Several studies have already demonstrated the successful application of iPSC technology for the generation of tumor-specific cytotoxic T lymphocytes (i-CTLs) and i-iNKT cells [22–28]. To generate iNKTbased antitumor immunotherapies, special interest has been paid to maintaining or expanding the CD4^-^ subpopulation [29], because this subset produces mainly TH1 cytokines and has a more cytolytic profile that corresponds to higher anti-tumor activity in vivo [30, 31]. In contrast, the CD4^+^ subset of iNKT cells appears more polyfunctional, producing pro-inflammatory cytokines such as IFN-γ and TNF-α, regulatory cytokines such as IL-4, IL-10, IL-13, and pro-hematopoietic cytokines such as GM-CSF and IL-3 [30]. Importantly, CD4^+^ iNKT cell production of pro-hematopoietic cytokines played a key role in their ability to promote hematopoiesis and hematopoietic engraftment [5, 6, 32]. These findings underscore the potential immunotherapeutic value of pro-hematopoietic iNKT cells derived from the CD4^+^ subset. However, the capacity of i-iNKT cells to support hematopoiesis has never been tested.

Here, we present the successful generation of iPSCs from CD4^+^ Vα24^+^ iNKT cells and a novel protocol for the efficient production of CD34^+^ hematopoietic progenitor cells from i-iNKT cells by applying a differentiation platform to enhance the generation of arterial hemogenic endothelium under fully defined conditions. As we reported previously, activation of arterial program through upregulation of NOTCH signaling, ETS1 or SOX17 overexpression, or modulation of MAPK/ERK signaling during PSC differentiation promotes the production of T cells [33–35]. In this study, we used the culture of hPSC spheroids on collagen IV with an optimized concentration of small molecule morphogenesis modulators to enhance the production of DLL4^+^CXCR4^±^ hemogenic endothelium with arterial features and progenitors with T-lymphoid potential. Using this protocol, we successfully regenerated i-iNKT cells and demonstrated their capacity to produce hematopoiesis-promoting cytokines and support the expansion of myeloid progenitors following stimulation.

## Results

### Generation of iPSCs from Human iNKT Cells

**Fig. 1:**
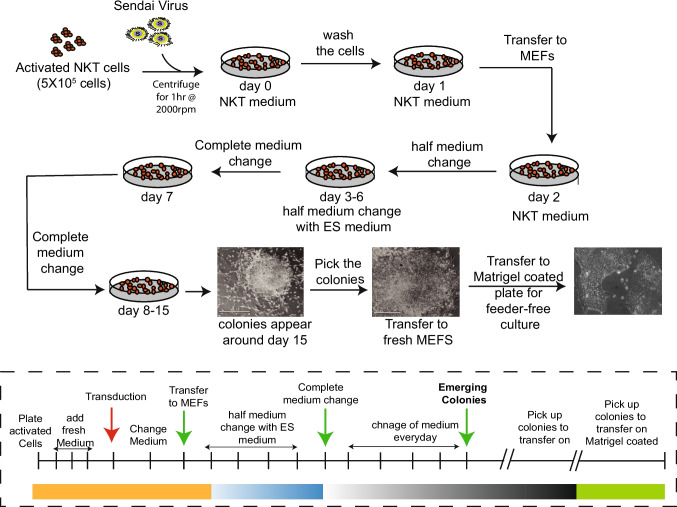
Schematic diagram of iNKT reprogramming and generation of iNKT-iPSCs

We have successfully developed and validated a highly efficient and reproducible method to reprogram CD4^+^ iNKT cells. Human CD4^+^ iNKT cells were sorted using human CD1d tetramers loaded with a lipid analogue of α-GalCer, expanded in vitro*,* and used for reprogramming after they had exited from log-phase proliferation. Following restimulation, CD4⁺ iNKT cells were reprogrammed into induced pluripotent stem cells (iPSCs) using the Cyto-Tune iPS2.0 Sendai reprogramming kit (Fig. 1). After reprogramming, individual cell colonies were picked and cultured on Matrigel-coated plates to maintain cells under feeder-free conditions in E8 medium/mTeSR plus medium. iPSC colonies generated from iNKT displayed typical pluripotent stem cell morphology and demonstrated normal karyotype and the expression of OCT4, SOX2, and NANOG by immunostaining (Figure S1A-F). To confirm the iPSC origin from iNKT cells, we analyzed TRAV10-TRAJ18 (Vα24-Jα18) gene rearrangements by PCR and confirmed that all the clones except clone 5 captured TCR rearrangement typical for iNKT cells (Figure S1E). Based on all these parameters, we concluded that reprogrammed iPSC clones represent human iNKT cell-derived iPSCs (iNKT-iPSCs).

### Generation of CD34+ hematopoietic progenitors using the culture of 3D spheroids on collagen IV

**Fig. 2:**
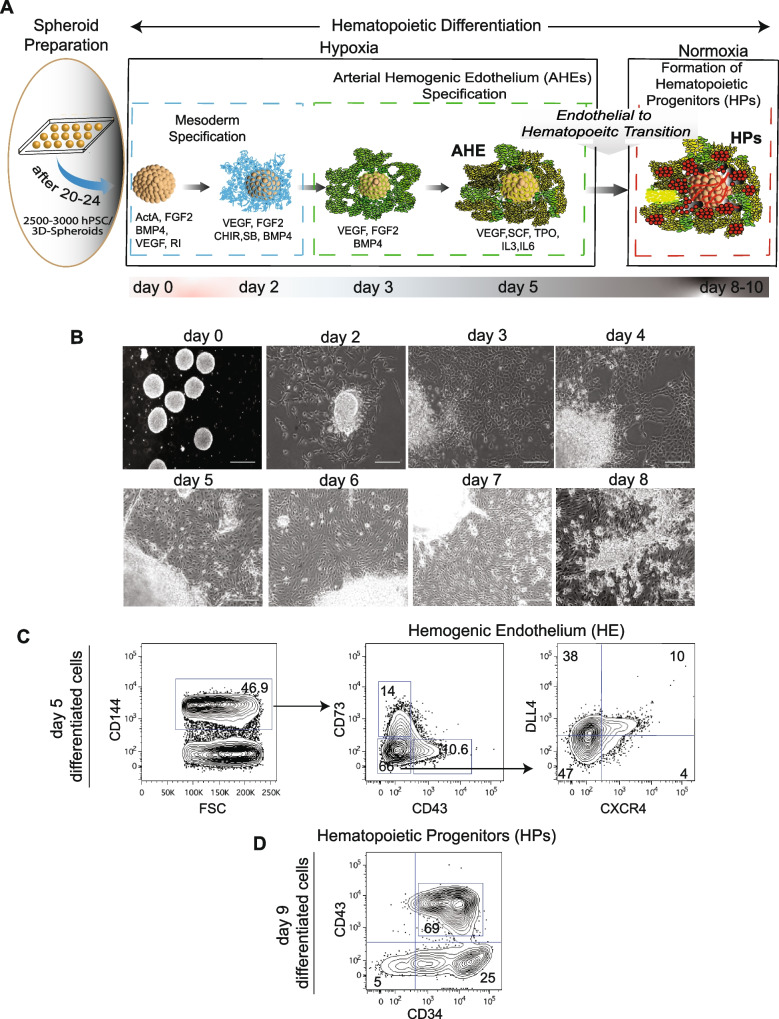
3D Spheroid hanging drop method for hematopoietic differentiation of iPSCs. **A** Schematic diagram of a 3D spheroid protocol used to generate CD34^+^ hematopoietic progenitors from iNKT-iPSCs. **B** Images of differentiating cultures reveal the outgrowth of mesodermal cells on days 2–3, the formation of hemogenic endothelium on days 4–5, and the expansion of round blood cells on day 8 of differentiation (scale bar, 50 μm). **C** Phenotypic characterization of hemogenic endothelium on day 5 of differentiation and **D** floating hematopoietic progenitors on day 9 of differentiation

To enhance the efficacy of generating CD34^+^ hematopoietic progenitors, we developed feeder-free, defined conditions for inducing blood formation from iPSC spheroids cultured on collagen IV. We used a low-cost medium to prepare the spheroid (75–150 μm) in a short period of time (12–24 h) using the “hanging drop” method. This method enables control over spheroid size by adjusting the volume of the drop (30–40 μL) or the density of the cell suspension (2,500–3,500 cells). We used 3000 iNKT-iPSCs to prepare a single spheroid. Following plating 140–160 spheroids per 50–60 cm^2^ surface area of plates coated with collagen IV, we differentiated them in the presence of hematopoietic cytokines and morphogens, including CHIR99021 and SB-431542 added at day (D2) of differentiation (Fig. 2A). Following adherence, we observed the outgrowth of mesodermal cells on days 2–3 of differentiation, and subsequently, endothelial cell formation and expansion occurred from day 4 to day 8 of differentiation. Hematopoietic progenitors budding from the hemogenic endothelium were first noticed starting from day 5 or 6 of differentiation and became abundant on day 8 of differentiation (Fig. 2B). Since our prior studies demonstrated that activation of the arterial program in CD144^+^CD43^−^CD73^−^ hemogenic endothelium (HE) increases the output of T cells [35–37], we optimized concentrations of WNT-signaling inhibitor, CHIR99021, and TGFβ-signaling inhibitor, SB-431542, to increase the formation of DLL4^+^CXCR4^±^ hemogenic endothelium with arterial features up to 50% (Fig. 2C). More than 90% of floating hematopoietic progenitors collected on day 9 of differentiation expressed CD34, with up to 70% of cells co-expressing the hematopoietic marker CD43 (Fig. 2D).

### Generation of i-iNKT from iNKT-iPSCs

**Fig. 3:**
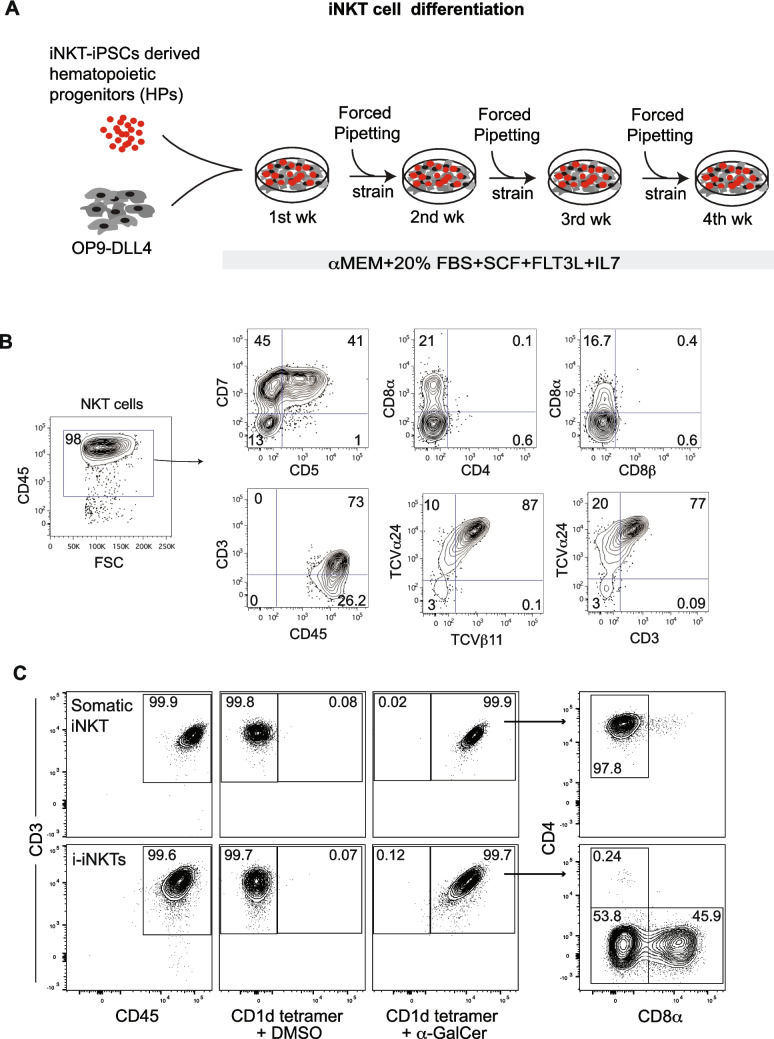
Generation of i-iNKT cells from iNKT-iPSCs. **A** Schematic diagram of differentiation protocol. **B** Phenotype of i-iNKT cells. **C** Binding of α-GalCer loaded CD1d tetramer to somatic iNKT and i-iNKT cells. Both somatic and i-iNKT cells are uniformly stained by α-GalCer loaded CD1d tetramer; i-iNKT cells have down-regulated CD4 and upregulated CD8α

We used our established T cell differentiation protocol [33, 38–41] to generate i-iNKT cells from CD34^+^ cells in coculture with feeders (Fig. 3A). We collected CD34^+^ hematopoietic progenitors from day 9 differentiation cultures (Fig. 2D) and replated them onto OP9 expressing human NOTCH ligand DLL4 (OP9-DLL4). In this co-culture, CD7^+^CD45^+^ lymphoid progenitors began to emerge by week 2 of coculture. By week 3–4, most of the cells in cultures (~ 87%) were TCVα24^+^TCVβ11^+^ double-positive (Fig. 3B). Despite being generated from reprogrammed CD4^+^ iNKT cells, the generated i-iNKT cells lacked expression of CD4 (Fig. 3B). A fraction of the cells expressed CD8α, but CD8β was not expressed (Fig. 3B).

After further culture in medium containing 200U IL-2 for an additional 7 days, nearly 100% of the generated i-iNKT cells expressed CD45 and CD3 at levels similar to an in vitro culture of somatic iNKT cells (Fig. 3C). Moreover, they similarly bound human CD1d tetramer loaded with the antigenic lipid α-galactosylceramide (α-GalCer) but were not stained by CD1d tetramer that was not loaded with a specific lipid (Fig. 3C). CD4 was not upregulated upon further culture of the i-iNKT cells, although CD8α expression appeared slightly increased (Fig. 3C). Overall, this analysis demonstrated that the TCR of the generated i-iNKT cells retained the ability to recognize a specific antigenic lipid presented by CD1d.

### Transcription Factor Profile and Cytokine Production by iNKT cells

**Fig. 4:**
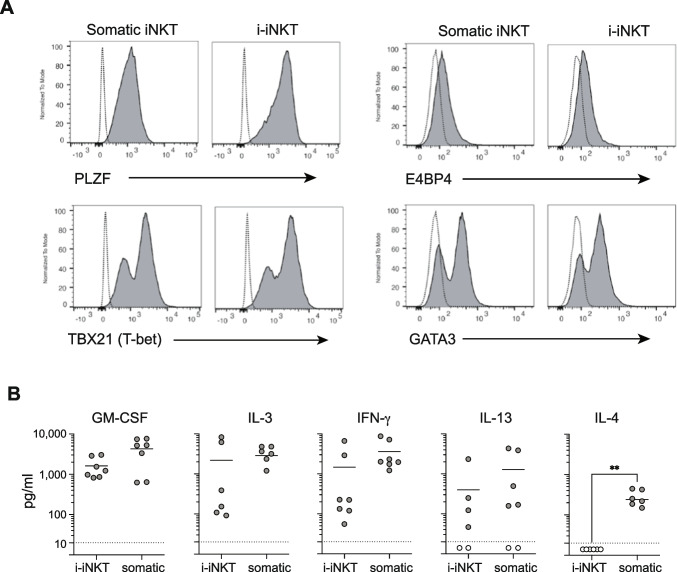
Functional characterization of i-iNKT cells. **A** Flow cytometric analysis of expression of key transcription factors in somatic iNKT and i-INKT cells. **B** Evaluation of cytokine production after anti-CD3 stimulation using ELISAs. Points colored in white under the dotted line of each graph represent data under the limit of detection for the respective ELISA

We next performed intracellular flow cytometric staining to assess i-iNKT cell expression of key transcription factors compared to somatic CD4^+^ iNKT cells. Like somatic iNKT cells, the i-iNKT cells were uniformly positive for PLZF, suggesting innate-like functional characteristics may be maintained (Fig. 4A). Additionally, the i-iNKT and somatic iNKT cells showed similar expression of transcription factors that govern cytokine production capacity. Specifically, they appeared nearly identical to somatic CD4^+^ iNKT cells for expression of T-bet (IFN-γ), E4BP4 (IL-13), and GATA-3 (IL-4).

To further investigate, we directly tested their TCR-dependent cytokine secretion. i-iNKT and somatic iNKT cell cultures were stimulated in parallel using anti-CD3 antibodies or were mock-treated (no stimulation), and culture supernatant was assayed by ELISA to assess levels of GM-CSF, IL-3, IFN-γ, IL-13, and IL-4. Whereas unstimulated cultures produced little or no detectable secretion of any of the cytokines, TCR stimulation consistently induced robust production of GM-CSF by i-iNKT cells at levels similar to those of somatic iNKT cells (Fig. 4B). Similar to somatic CD4^+^ iNKT cells, the i-iNKT cells also clearly produced IL-3, IFN-γ, and IL-13 (Fig. 4B). However, in contrast to the somatic iNKT cells, the i-iNKT cells showed no detectable secretion of IL-4 (Fig. 4B).

### i-iNKT Cells Demonstrate Pro-Hematopoietic Activity

**Fig. 5:**
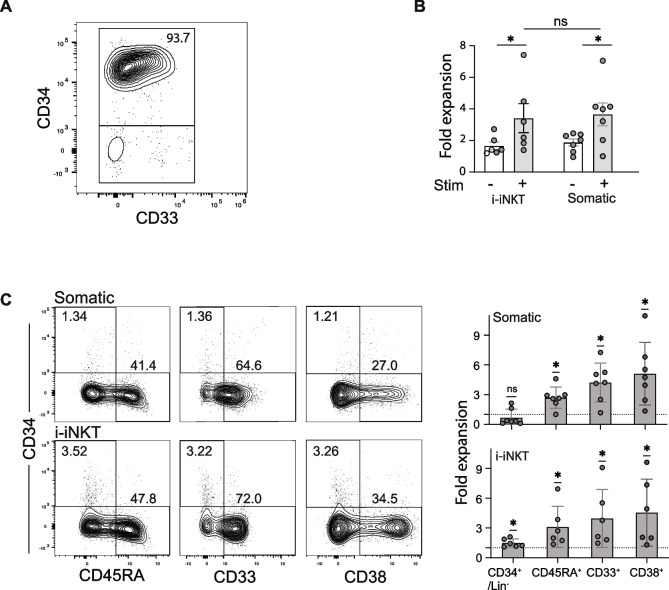
Hematopoiesis supportive activities of i-iNKT cells. **A** Phenotype of magnetically sorted human umbilical cord blood CD34^+^ cells used in HSC differentiation experiments. Starting populations were 90–93% pure. **B** Fold expansion of total cells after 14 days of culture with 7 days of exposure to i-iNKTS or somatic iNKTs. The cell number is expressed as a fold increase over the minimal medium condition. **C** Phenotype of CD34^+^ and CD34^−^ cells after 14 days of culture with 7 days of exposure to i-iNKTS or somatic iNKTs. The *p*-values of less than 0.05 are denoted with an asterisk *

Seeing that i-iNKTs maintained the ability to produce GM-CSF and IL-3, we investigated if they could promote hematopoietic differentiation and expansion similarly to somatic iNKT cells. We used magnetic sorting to purify CD34^+^ from human umbilical cord blood samples, resulting in a population that was highly enriched for CD34^+^ cells (Fig. 5A). These were cultured in the bottom wells of transwell plates alone, or in the presence of resting or anti-CD3 stimulated somatic iNKTs or i-iNKTs in the transwell insert. All conditions were cultured in a serum-free media with a "minimal" cytokine mix (SCF, TPO, FlT3L, and IL-7) that maintains viability but produces only suboptimal differentiation and expansion of CD34^+^ hematopoietic stem/progenitor cells (HSPCs). Transwell inserts containing i-iNKT or somatic iNKT cells were removed after seven days of culture. After an additional 7 days of culture (14 days total), we harvested the cells from the lower wells and assessed whether the presence of i-iNKT cells led to enhanced expansion or differentiation.

Since there was substantial variation in hematopoietic activity among CD34^+^ cells isolated from different human umbilical cord blood samples, we normalized the assay results by assessing fold change compared to cells cultured in parallel in minimal cytokine mix alone. Exposure to transwell inserts containing anti-CD3 activated i-iNKT or somatic iNKT cells resulted in significantly increased total cell numbers compared to cells cultured in the presence of their unstimulated counterparts, and there was no significant difference between activated i-NKT and somatic iNKTs in the amount of cellular expansion induced in the lower transwells (Fig. 5B).

To determine whether the cellular expansion resulted from HSPC proliferation or differentiation, we performed flow cytometry on the lower well cells for CD34 and lineage differentiation markers such as CD45RA, CD33, and CD38. Stimulated i-iNKT and somatic iNKT cells both significantly increased the fold-expansion of cells that lost CD34 and gained lineage markers compared to minimal media conditions (Fig. 5C). There was no notable difference in the fold-expansion of differentiating cells between somatic and i-iNKT cell stimulation. Interestingly, compared to culture in minimal cytokine medium alone, exposure to activated i-iNKT cells—but not somatic iNKT cells—led to a significantly higher number of cells that retained CD34 and were negative for lineage markers (Fig. 5C). Collectively, these findings indicate that activated i-iNKT cells produce secreted factors similar to those of somatic iNKT cells, which promote hematopoietic differentiation and expansion, and suggest that i-iNKT cells may be more capable of supporting HSPC self-renewal.

## Discussion

In this study, we developed a protocol for generating iPSCs from somatic iNKT cells and differentiating them into i-iNKT cells with hematopoiesis-supporting activities. In contrast to prior studies [22, 23], we generated i-iNKT iPSCs using reprogramming vectors without SV40 large T cell antigen, which interferes with DNA damage and repair mechanisms and may cause genomic instability. In addition, we established a 3D spheroid protocol for the efficient generation of CD34^+^ hematopoietic progenitors by enhancing the production of arterial hemogenic endothelium with strong T cell potential. In coculture with OP9-DLL4, iCD34^+^ cells produced i-iNKT cells with a typical TCR Vα24^+^Vβ11^+^ that bound to CD1d molecules loaded with α-GalCer. These i-iNKTs also expressed PLZF, the signature transcription factor of iNKT cells, suggesting they possess similar innate-like characteristics. Additionally, the i-iNKT cells showed similar expression of other master-regulator transcription factors (T-bet, GATA-3, and E4BP4) and produced a similar cytokine profile to that of their somatic counterparts. Most significantly, stimulated i-iNKT cells secreted factors that increase the expansion and degree of differentiation of myeloid progenitors. Key to this activity is that the i-iNKTs produced the cytokines GM-CSF and IL-3, however, other secreted factors may also be at play.

The pro-hematopoietic cytokine production exhibited by i-iNKT and somatic iNKT cells is consistent with their unusual transcription factor expression profile, where T-bet and GATA-3 are both upregulated, as T-bet has previously been shown to promote GM-CSF production by human T cells [42] and GATA-3 induces IL-3 production [43, 44]. Expression of T-bet is also consistent with their production of IFN-γ, while GATA-3 may confer the capacity to produce IL-13. Notably, both i-iNKT and somatic iNKT cells also showed detectable expression of the E4BP4 transcription factor. Prior studies have shown that this transcription factor induces production of IL-10 and IL-13 by conventional T cells, and is required for IL-10 production by murine iNKT cells [45, 46]. However, although we have previously observed that a fraction of primary CD4+^+^ iNKT cells in human blood can produce IL-10 [30], we found that neither the i-iNKTs nor the somatic iNKT cells used in these analyses consistently produced IL-10 (data not shown). Since E4BP4-expressing murine iNKT cells that produce high levels of IL-10 have been shown to activate regulatory pathways that lead to control of inflammatory pathology in vivo [45] it will be of interest in future studies to explore whether strategies to augment E4BP4 expression levels in human i-iNKT cells result in a cell population that is optimized for therapeutic applications requiring anti-inflammatory activity.

While the pro-hematopoietic activity of i-iNKTs and somatic iNKTs appears largely similar, there were differences in other characteristics, mainly the failure of the i-iNKTs to express CD4 and IL-4. Previous groups that have generated iPSC-derived iNKTs also saw a deficiency in IL-4 production [22, 23]. One group that produced iPSC-derived i-iNKTs from CD4^+^ iNKT cells noted that though their i-iNKTs displayed a CD4/CD8 double-negative (DN) surface phenotype, they still had a functional profile similar to CD4^+^ iNKT cells in that they produced both TH1 and TH2 cytokines, and had gene expression profiles similar to CD4 iNKTs, but genes for IL-4 and CD4 were hypermethylated [22]. Thus, our i-iNKTs might also have methylated IL-4 and CD4 genes, as this methylation could be a byproduct of the iPSC re-differentiation process. It will thus be of interest in future studies to investigate whether DNA methylation editing could potentially restore the ability of i-iNKTs to produce IL-4.

Further analysis will also be required to assess functional outcomes of i-iNKT cell interactions with key CD1d^+^antigen presenting cells (APCs) such as monocytes and dendritic cells (DCs). Our prior studies have shown that somatic iNKT cell recognition of self lipids presented by CD1d^+^ APCs preferentially induces secretion of GM-CSF, while strong TCR signaling such as that induced by the synthetic ligand α-GalCer amplifies secretion of both IFN-γ and IL-4 [47]. In contrast, signals from the pro-inflammatory cytokine IL-12p70 or from binding of LFA-1 on iNKT cells to ICAM-1 (an adhesion ligand that is typically expressed at comparatively high levels on cells such as monocytes and DCs) selectively enhance iNKT cell production of IFN-γ [47, 48]. We used anti-CD3 antibody stimulation to activate cytokine production by i-iNKT cells in this study, which provides comparatively strong TCR stimulation without co-stimulatory signals that are likely to alter iNKT cytokine profiles. It will thus be important in future studies to establish how TCR stimulation from CD1d^+^ APCs in the presence or absence of co-stimulatory signals impacts cytokine secretion by i-iNKT cells.

In the hematopoietic differentiation experiments performed here, we used a transwell culture system to avoid potential artifacts from exposing the purified CD34^+^ HSPCs to antibody-coated beads. However, in a prior study Kitayama et al. reported that redifferentiated i-iNKT-cells can exhibit NK-like cytotoxic activity [22] raising the concern that direct contact between HSPCs and i-iNKT cells could result in cytolytic effects that could limit hematopoietic differentiation and expansion. Importantly, we did not observe evidence of significantly reduced viability in an experiment where HSPCs were directly co-cultured with i-iNKT cells (Figure S2), suggesting that exposure to HSPCs does not activate cytotoxicity. Consistent with this, we have previously found that unless they receive a strong TCR signal (e.g. via recognition of α-GalCer) somatic iNKT cells exhibit little evidence of cytotoxic activity [49]. Moreover, we found that CD4^+^ somatic iNKT cells profoundly enhanced hematopoietic engraftment in a xenotransplant model in vivo [7], suggesting that their potential for cytolytic activity is not a limiting factor in this context.

By demonstrating the capacity of i-iNKT cells to produce pro-hematopoietic effects, our study supports the potential for developing i-iNKTs as an off-the-shelf therapeutic modality during hematopoietic stem cell transplantation (HSCT). HSCT is the preferred treatment for various hematological diseases, including blood cancers and severe immunodeficiency. Patients must undergo an intense chemotherapy conditioning to ablate any cancer cells (in the case of malignant disease) and their own immune system before having hematopoietic stem cells transplanted to reconstitute a new, disease-free immune system. However, successful engraftment of transplanted stem cells, preventing graft versus host disease (GVHD), and promoting graft versus tumor effects after HSCT remains a significant clinical hurdle in the treatment of blood cancers. Clinical data have shown that HSCT grafts containing higher than average ratios of iNKT cells are associated with a lower risk of acute GVHD [50]**.** Additionally, the maintenance of a higher ratio of iNKT cells to T cells post-transplant also predicted lower rates of acute GVHD and mortality. The mechanisms underlying the beneficial effects of iNKT cells in HSCT patients may vary depending on the iNKT subset. CD4 expression distinguishes two functionally distinct subsets of human iNKTs, with the CD4-negative subset responsible for most cytotoxic activity and showing a TH1-biased cytokine profile, and the CD4^+^ subset showing a polyfunctional profile that includes both pro-inflammatory and regulatory activities. CD4^−^ iNKT cells may thus prevent acute GVHD by lysing DCs that activate pathogenic T cells, whereas CD4^+^ iNKT cells appear to protect against GVHD by limiting alloreactive T cell activation through production of regulatory cytokines that promote expansion of donor Tregs [50–53]**.** While both CD4^+^ and CD4^−^ iNKTs can reduce a T cell alloreactive response, the CD4^+^ iNKT mechanism appears to preserve the donor T cell-mediated graft-versus-tumor response [51, 53]. Thus, a key area for future studies will be to determine whether i-iNKT cells derived from the CD4^+^ somatic iNKT lineage are also capable of reducing GVHD risk, while maintaining donor T cell anti-tumor responses, and promoting hematopoietic engraftment.

## Materials and Methods

### Reprogramming of iNKT Cells

Activated iNKT cells were maintained in the NKT medium consisting of RPMI 1640, 10% heat-inactivated fetal bovine serum (GeminiBio), 5% heat-inactivated defined bovine calf serum (Hyclone), 3% human AB serum (Atlanta Biologicals), 1 × L-glutamine, and cytokine IL2 (200U/ml). iNKT cells were reprogrammed using the CytoTune™-iPS 2.0 Sendai Reprogramming Kit (ThermoFisher Scientific) to generate iNKT-iPSCs. The scheme of generating iPSCs is shown in Fig. 1. For reprogramming, 0.5 × 10^6^ activated live cells were transduced by the Klf4, Oct4, and SOX2 (KOS) virus, and the c-Myc virus at a multiplicity of infection (MOI) of 5 (KOS MOI = 5, hc-Myc MOI = 5), and the Klf4 virus at an MOI of 3 (hKlf4 MOI = 3) in a total volume of 0.5 ml of NKT medium. The day after transduction (Day 1), Sendai viruses were removed by spinning down the cell suspension. The cells were resuspended in fresh complete NKT medium and plated in a 24-well plate for an extra day. On day 2, the cells were transferred onto mouse embryonic fibroblasts (MEFs). On day 3, the transition into iPSC medium began by replacing half of the NKT medium with human ES medium. From day 7 on, the full volume of medium was changed to ES Medium. The medium was replaced every day thereafter, and the emergence of iPSC colonies was monitored. Colonies began to appear around day 15 after transduction, and after 1–2 weeks, colonies were ready for transfer. The colonies were manually picked and transferred onto fresh MEFs for expansion and characterization. Single colonies were also picked and cultured on Matrigel-coated plates for feeder-free expansion. The iPSC lines (iNKT-iPSCs) were maintained on MEFs or Matrigel-coated plates in E8 or mTeSR1plus medium. Cells were passaged every 3–4 days using Collagenase Type IV (Life Technologies) for MEFs and EDTA (0.5 mM) for feeder-free culture.

### Immunofluorescence

The expression of pluripotency markers was analyzed by immunofluorescence. Briefly, cells were fixed for 15 min, washed with phosphate-buffered saline (PBS), permeabilized with a solution of 0.1% Triton-X (Sigma) for ten minutes, washed with PBS, and incubated overnight with anti-human OCT3/4 (1:200; Santacruz Biotechnology), anti-human SOX2 (1:400; Cell Signaling), and anti-human NANOG (1:250; Cell Signaling). Cells were washed five times with PBS-Tween 20 (PBST) and incubated with anti-mouse Alexa 555 conjugated (1:1000; Secondary antibodies, Invitrogen) or anti-rabbit IgG Alexa Fluor 488 conjugate (1:1000; Secondary antibodies, Invitrogen) for one hour, washed with PBST, and incubated with DAPI (1:1000; Sigma) for 10 min. The immunolabeled cells were examined using the Nikon Eclipse Ti-E confocal system (Nikon Instruments Inc).

### Detection of Human iNKT-Specific TCR Rearrangement in iNKT-iPSCs

Genomic DNA was purified using a Zymo Cell Kit according to the manufacturer’s instructions. Purified DNA was subjected to PCR to detect the Vα24 + iNKT cell-specific rearranged TCR using the primers 5’- AGCACTGGGAGAGGTCCTGTTTC −3’ and 5’- GAATAATGCTGTTGTTGAAGGCG-3’.

### Hematopoietic Differentiation of hPSCs

The iNKT-iPSCs were differentiated into CD34^+^ hematopoietic progenitors using a 3D spheroid differentiation protocol. Briefly, on day −1, iPSCs were singularized and resuspended in an organoid medium containing 2.6% methylcellulose, 10% BIT serum substitute (StemCell Technologies) in IF9S differentiation medium[39]. Small droplets of 30 μl were made on square dishes such that each spheroid contained about 3000 cells, and the plate was closed and placed in an inverted position overnight in the incubator so that the cells could aggregate in the drop by the hanging drop method. The following day (day 0), the spheroids were harvested with phosphate-buffered saline or iPSC medium, centrifuged, and resuspended in differentiation medium supplemented with BMP4, VEGF, FGF (25-50ug/ml), Activin A (5–15 ng/ml) and ROCK inhibitor (1–10 μM) and plated on collagen IV coated plates and cultured in hypoxia (5% O_2_, 5% CO_2_). On day 2, the media was changed to differentiation medium supplemented with VEGF, FGF (25–50 ng/ml), BMP4 (1–10 ng/ml), CHIR99021 (1–3 μM), and SB-431542 (2–5 μM), and the cells were placed back in the hypoxia incubator. On day 3, the medium was replaced with differentiation medium supplemented with VEGF, FGF (25–50 ng/ml), BMP4 (1–10 ng/ml), and placed back in the hypoxia incubator. On Day 5, the medium was replaced with differentiating medium supplemented with SCF, VEGF, TPO, IL6 (25–50 ng/ml), FGF, IL3 (1–10 ng/ml), and the plates were placed in a normoxia incubator with 5% CO_2_. For some studies, the culture plates on Day 5 were sorted by fluorescence-activated cell sorting (FACS) to isolate D5 hemogenic endothelium subsets for further studies. On Day 7, an additional differentiation medium supplemented with SCF, VEGF, TPO, IL6 (25–50 ng/ml), FGF, and IL3 (1–10 ng/ml) was added. Floating progenitors or total CD34^+^ hematopoietic progenitors isolated by MACS were collected on days 9 or 10 for further study.

### Generation of i-iNKT Cells from iNKT-iPSCs

Schematic of i-iNKT cells differentiation from iNKT-iPSCs is shown in Fig. 3A. OP9-DLL4 cells were maintained in αMEM media containing 20% FBS on a 0.1% gelatin-coated 10 cm cell culture dish. For i-iNKT differentiation, OP9-DLL4 feeder layers were prepared in 6-well plates. The floating or total CD34^+^ hematopoietic progenitors isolated by MACS were collected from D9-10 of iNKT-iPSCs hematopoietic differentiation cultures, strained through a 70 mm cell strainer (ThermoFisher Scientific), and resuspended in a T cell differentiation medium consisting of αMEM (Gibco) supplemented with 20% HyClone™ FBS, 5 ng/ml IL-7 (Peprotech), 5 ng/ml Flt3-Ligand (Peprotech), and 10 ng/ml SCF (Peprotech). The cells (1 × 10^5^ cells/well of 6-well plate) were cultured on OP9-DLL4 at 37 °C and 5% CO2 for 4 weeks with weekly passages. Every 6–7 days, cells were collected by vigorous pipetting, filtered through a 40 μm cell strainer, and transferred onto a fresh OP9-DLL4 monolayer.

### iNKT Cell Culture

i-iNKTs were differentiated as previously described. Somatic iNKTS were clonal or polyclonal cell lines previously established by our group [54]. The somatic iNKT cell lines are primary, CD4^+^ iNKT cells isolated from human peripheral blood. Somatic iNKT cell lines were sorted from peripheral blood mononuclear cells using CD1d tetramer loaded with $\alpha$-GalCer. Cells were then expanded using irradiated PBMCs and 1 μg/ml phytohemagglutinin in culture medium consisting of RMPI 1640 with 10% heat-inactivated FBS, 5% heat inactivated BCS, 3% human serum, 1% L-glutamine, and with 200 U/ml recombinant human IL-2. Both somatic iNKT and i-iNKT lines were maintained in the same culture medium.

### Flow Cytometry

For antibody staining, cells were prepared in PBS containing 2% FBS, 1 mM EDTA, and 0.1% sodium azide and stained with antibodies listed in Supplemental Table S1. Expression of iNKT and T cell markers was evaluated following gating of CD45^+^ cells. Cell analysis was performed using MACSquant (Miltenyi Biotech) or an AttuneNxt Acoustic Focusing cytometer (Thermo Fisher Scientific), and the acquired data were analyzed by Flowjo software. For transcription factor staining, somatic iNKT cells and i-iNKT cells were stained with surface markers including CD3, CD4, CD8β, and 6B11. Then, the cells were fixed, permeabilized, and stained with antibodies against a specific transcription factor or negative control antibodies according to the manufacturer’s protocol using the BD Biosciences transcription factor buffer set.

### Loading of PE-Labeled CD1d Tetramers

PE-labeled CD1d tetramer from the NIH at 100 μg/ml concentration was mixed with a lipid analog of $\alpha$-GalCer at 40 μg/ml or an equivalent volume of DMSO. The lipid analog of $\alpha$-GalCer was sonicated for 45 min in a 37 °C water bath before being immediately added to the tetramer. $\alpha$-GalCer or DMSO-loaded tetramers were incubated overnight at 37 °C in an incubator and then titrated by flow staining to determine the optimal volume for flow staining.

### Cytokine Profiling of i-iNKTs

Hi-binding 96-well plates were coated with 2 μg/ml or 1 μg/ml anti-CD3 antibody overnight at 4 °C. Plates were washed with PBS and culture medium without IL-2. Somatic iNKTs or i-iNKTs in cell culture medium lacking IL-2 were added at 2.5 × 10^4^ cells/well and incubated at 37°. Supernatant was collected at 24 h and assayed for GM-CSF, IL-3, IFN-$y$, IL-13, and IL-10 using sandwich ELISA.

### Cord Blood CD34 + Enrichment

De-identified human umbilical cord blood samples were obtained from the Carolinas Cord Blood Bank or the Cleveland Cord Blood Center. Cord blood mononuclear cells (CBMCs) were isolated on a density gradient using Ficoll after diluting the cord blood 1:1 in PBS. CBMCs were purified for a second time on a Ficoll gradient if red blood cell contamination was present. CD34^+^ cells were labeled using CD34 Miltenyi beads and positively selected using a magnetic sort. CD34^+^ enriched cells were stained by flow to assess purity and were at least 90% pure in each experiment.

### HSC Expansion and Differentiation Assay

To test whether iNKTs can secrete factors that support hematopoietic differentiation and expansion, CD34^+^ enriched cells from cord blood were cultured either alone or in the bottom of a trans-well with resting or anti-CD3 stimulated i-iNKTs or somatic iNKTs in the upper trans-well insert. CD34^+^ cells were suspended in Xvivo-15 media seeded at 5–10 × 10^3^ cells per well. Somatic iNKTs or i-iNKTs were preincubated with anti-CD3/CD28 dynabeads or anti-CD3/CD28 Immunocult T cell activator reagent before being added to the upper trans-well inserts. Cells were cultured in “minimal media” designed to support suboptimal expansions and differentiation. Minimal media consisted of XVIVO-15 media supplemented with 20 ng/ml of Flt3L, TPO, SCF, and IL-7 (Peprotech). Wells were re-fed with the above cytokines every 3 days. Trans-well inserts containing iNKTs were removed at day seven. At day 14 of differentiation, the total number of cells in each well was counted using a hemocytometer to determine the effect of iNKTs on cell expansion. To assess markers of differentiation, cells were stained with fluorescently labeled antibodies from BioLegend for CD34, CD33, CD45RA, and CD38 and read by flow cytometry.

## Supplementary Information

Below is the link to the electronic supplementary material.

Supplementary file1 (PDF 1742 KB)

## Data Availability

No datasets were generated or analysed during the current study.
