# Supplementary material for: Generation of iPSC-Derived iNKT Cells with Pro-Hematopoietic Activity
Source: Stem Cell Rev Rep. 2025 Dec 11;22(2):693–706. doi: 10.1007/s12015-025-11031-2 (PMC12858510; doi:10.1007/s12015-025-11031-2)
Supplement: Supplementary file 1 — Supplementary file1 (PDF 1742 KB) [file 12015_2025_11031_MOESM1_ESM.pdf]

Supplementary Information (SI)
A. Kumar et al. "Generation of iPSC-Derived iNKT Cells with Pro-Hematopoietic Activity".

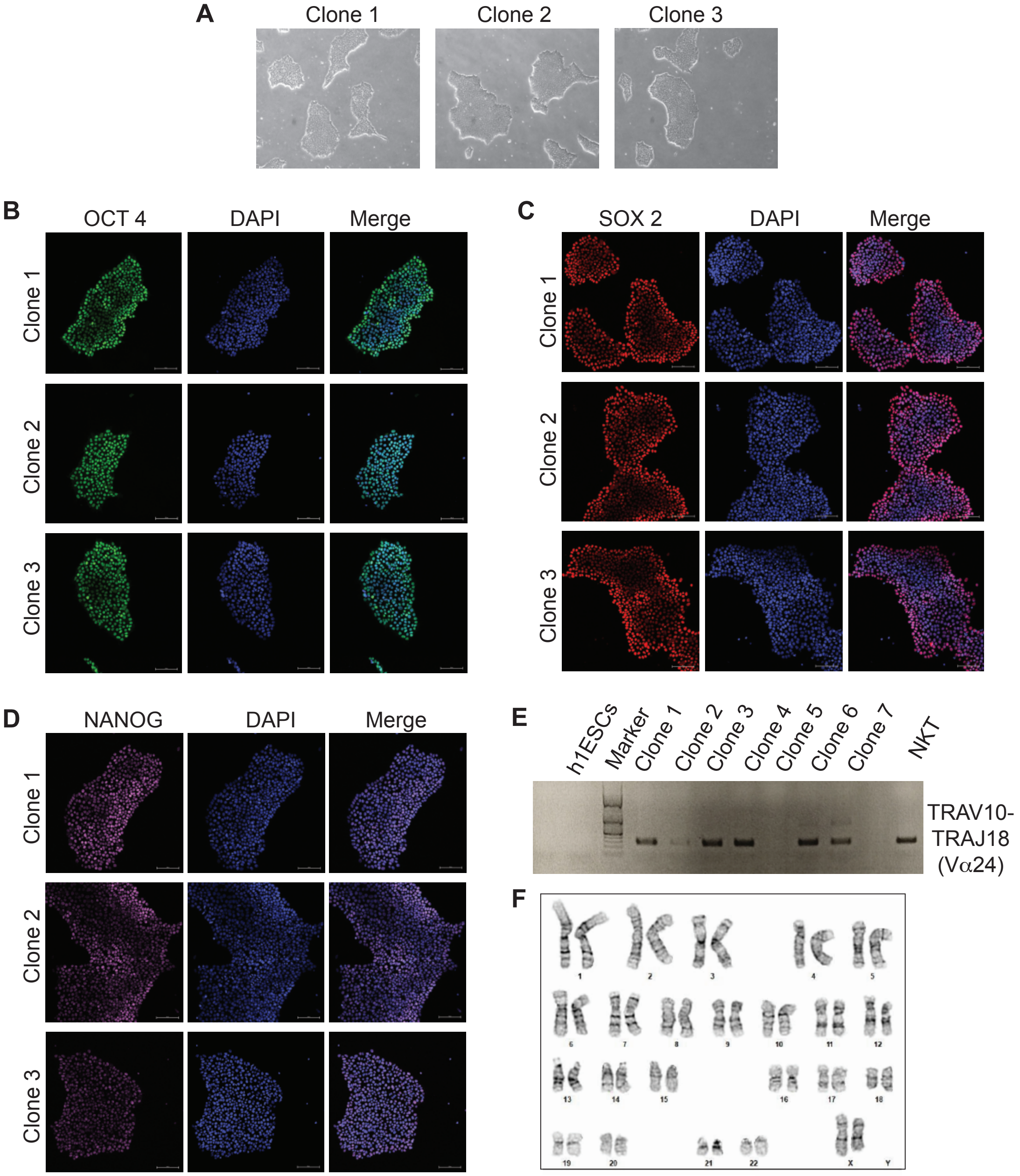

Supplementary Figure S1. Characterization of iPSCs derived from iNKT cells. (A) Morphology of iNKT-iPSC colonies. (B) Immunofluorescent analysis of the expression of pluripotency markers, OKT4, SOX2, and NANOG in iNKT-iPSCs. (E) Detection of iNKT-specific TCR rearrangement in iNKT-iPSC clones using genomic PCR. (F) Normal karyogram of the iNKT-iPSC clone 3.

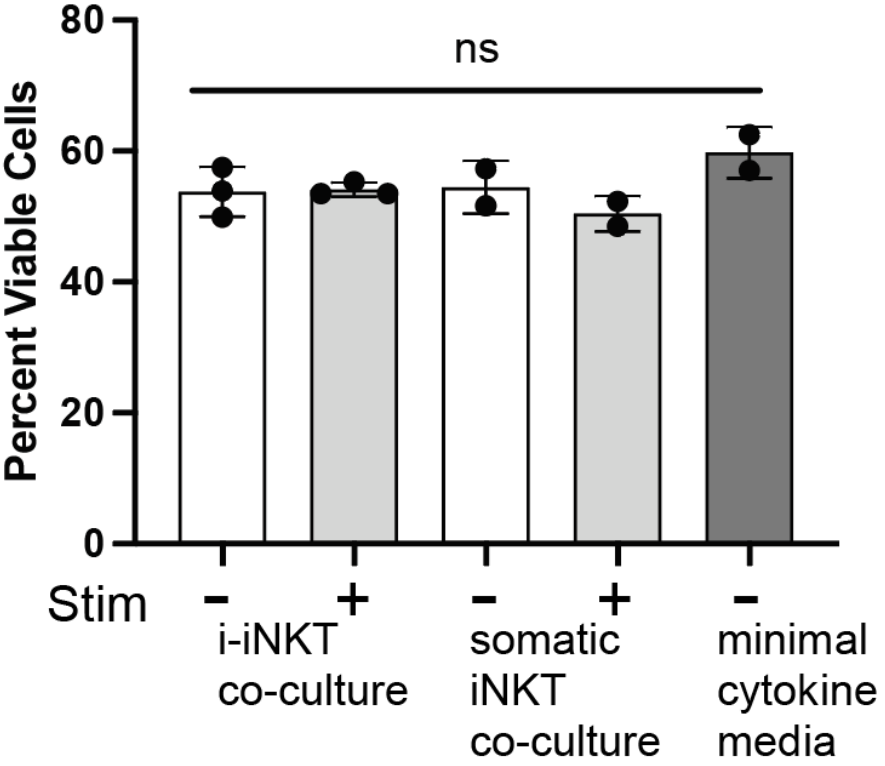

Supplementary Figure S2. Cellular viability is not significantly reduced following co-culture of HSPCs with i-iNKT cells. Purified $CD34^+$ HSPCs were cultured in XVIVO-15 serum-free media containing Flt3L, TPO, SCF, and IL-7 (”minimal cytokine medium”) alone or in contact with i-iNKT or somatic iNKT cells. Where indicated, the iNKT cells were stimulated by the addition of beads coated with anti-CD3 and anti-CD28 antibodies.

Supplementary Table S1: Antibodies used in the study

Antigen	Source	Clone	Identifier	RRID
CD3 APC	Biolegend	UCHT1	300412	AB_314066
CD3 AF700	BioLegend	HIT3a	300324	AB_493739
CD3 AF488	Biolegend	HIT3a	317310	AB_571877
CD4 PE	BD Biosciences	RPA-T4	555349	AB 398593
CD4	BioLegend	OKT4	357424	AB_2721519
CD5 PE	BD Biosciences	UCHT2	555353	AB_395757
CD7 PE Cy7	Miltenyi Biotec	CD7-6B7	564019	AB_2738545
CD8α PE	BD Biosciences	HIT8a	555635	AB_395997
CD8α FITC	BioLegend	SK1	344704	*AB_2566513*
CD8β PeCy7	eBioscience	SIDI8BEE	25-5273-42	AB_11219680
CD8β PE Vio770	Miltenyi Biotec	REA715	130-110-512	AB_2659528
CD34 PE	BD Biosciences	581	555822	AB_396151
CD43 APC	BD Biosciences	IG-10	560198	AB_1645460
CD73 BV421	BD Biosciences	AD2	562430	AB_11153119
CD144 PE	BD Biosciences	55-7H1	560410	AB_1645502
CD184	Miltenyi Biotec	REA649	130-116-521	AB_2727587
DLL4 PE Vio770	Miltenyi Biotec	MHD4-46	130-101-587	AB_2651569
TCRVβ11 APC	Beckman Coulter	C21	A66905	AB_3683578
TCRVα24 PC7	Beckman Coulter	C15	A66907	
TCR Vα24-Jα18	Biolegend	6B11	342916	AB_2721323
CD38 PE	BioLegend	HB-7	356604	AB_2561900
CD33 APC cy 7	BioLegend	P67.6	366613	AB_2566415
CD34 AF647	BioLegend	8G12	343508	AB_1877133
CD10 FITC	BioLegend	HI10A	312208	AB_314919
CD45	BioLegend	HI30	304044	AB_2563812
CD45RA	BioLegend	HI100	304136	AB_2563653
CD1d Tetramer PE	NIH			
Anti-PLZF	eBiosciences	Mags.21F7	12-9320-82	AB_11148934
E4BP4(NFIL3)	eBiosciences	MABA223	51-9812-42	AB_11148934
GATA3	Biolegend	16E10A23	653810	AB_2563217
T-bet	Biolegend	4B10	644810	AB_2200542
GM-CSF Capture	BioLegend	BVD2-23B6	502202	AB_315212
GM-CSF Detection	BioLegend	BVD2-21C11	502304	AB_315218
IL-3 Capture	BioLegend	BVD8-3G11	500502	AB_315103

IL-3 Detection	BioLegend	BVD3-1F9	500604	AB_2123713
IFN-γ Capture	BioLegend	MD-1	507502	AB_2122339
Biotin IFN-γ Detection	BioLegend	4S.B3	502504	AB_315229
IL-13 Capture	BioLegend	JES10-5A2	501902	AB_315197
Biotin IL-13 Detection	BioLegend	Poly5020	502001	AB_2124285
IL-4 Capture	BioLegend	8b4-8	500702	AB_315114
Biotin IL-4 Detection	BioLegend	MP4-25D2	500804	AB_315123
